# Prognostic Role of High-Sensitivity C-Reactive Protein/Albumin Ratio in Heart Failure Patients

**DOI:** 10.3390/biomedicines14040748

**Published:** 2026-03-25

**Authors:** Domenico Martire, Giuseppe Armentaro, Giandomenico Severini, Carlo Alberto Pastura, Maria Rosangela Scarcelli, Velia Cassano, Martina Crasà, Ilaria Gareri, Gianluca Cortese, Valentino Condoleo, Raffaele Maio, Giorgio Sesti, Francesco Andreozzi, Angela Sciacqua

**Affiliations:** 1 Department of Medical and Surgical Sciences, University “Magna Graecia” of Catanzaro, 88100 Catanzaro, Italymartinacrasa@gmail.com (M.C.);; 2 Geriatrics Division, “Renato Dulbecco” University Hospital of Catanzaro, 88100 Catanzaro, Italycondoleovalentino@gmail.com (V.C.); raf_maio@yahoo.it (R.M.); 3 Department of Clinical and Molecular Medicine, University of Rome-Sapienza, 00189 Rome, Italy; giorgio.sesti@uniroma1.it; 4 Research Center for the Prevention and Treatment of Metabolic Diseases (RC-METDIS), University “Magna Graecia” of Catanzaro, 88100 Catanzaro, Italy

**Keywords:** heart failure, inflammation biomarkers, hs-CRP/serum albumin ratio, MACE

## Abstract

The ratio of high-sensitivity C-reactive protein (hs-CRP) to Serum albumin (SA) (hs-CRP/SA) is emerging as a new potential biomarker capable of stratifying cardiovascular risk in patients with chronic HF (CHF), particularly the risk of major adverse cardiovascular events (MACEs). **Objectives**: The aim of this study was to evaluate the long-term prognostic value of the hs-CRP/SA ratio on the risk of MACEs in a population of outpatients with CHF. **Methods**: In this retrospective observational study, 500 patients were enrolled and were stratified into two groups based on the median value of the hs-CRP/SA ratio: 249 patients with hs-CRP/SA < 1.19 and 251 patients with hs-CRP/SA ≥ 1.19. **Results**: During median follow-up of 5.2 years, 3.6 MACEs/100 patients/year were detected; patients with hs-CRP/SA ≥ 1.19 had a MACE incidence of 5.9 events per 100 patient-years, compared with 1.2 events per 100 patient-years in those with hs-CRP/AS < 1.19 (*p* < 0.001). Multivariate analysis confirmed that hs-CRP/SA ≥ 1.19 was associated with an approximately 6.5-fold increased risk of new MACEs (HR 6.513, 95% CI 3.928–10.797; *p* < 0.001). **Conclusions**: The hs-CRP/SA ratio is confirmed as a powerful prognostic marker in patients with CHF, associated with a significantly increased risk of MACEs.

## 1. Introduction

Heart failure (HF) is a complex and constantly evolving clinical syndrome, characterized by the heart’s inability to ensure adequate peripheral perfusion in relation to the body’s metabolic demands. It is a condition with a high epidemiological impact, with a prevalence that, in Western countries, exceeds 2% in the adult population, reaching up to 10% in subjects over 70 years of age [1]. The progressive aging of the population and the increase in survival after myocardial infarction and other cardiovascular diseases have contributed to a constant increase in its incidence. HF is now one of the main causes of hospitalization and rehospitalization, especially in the elderly population, with a consequent significant economic impact on health systems [2]. From a clinical point of view, HF is configured as a heterogeneous syndrome both in etiology and clinical manifestations, requiring a multidimensional diagnostic–therapeutic approach [3]. In recent decades, pharmacological and technological innovations have radically changed the management of the disease, with the introduction of new therapeutic classes, including sodium–glucose cotransporter 2 inhibitor (SGLT2i) and angiotensin receptor–neprilysin inhibitor (ARNi), which have improved long-term outcomes, especially in patients with reduced ejection fraction [4]. However, mortality and morbidity rates associated with HF remain high, demonstrating the need to improve risk stratification tools and personalize therapy [5]. In this context, early identification of HF clinical phenotypes plays a central role. Current European and United States guidelines propose a classification into three subgroups based on left ventricular ejection fraction (LVEF): heart failure with reduced ejection fraction HFrEF: LVEF < 40%; HF with mildly reduced EF (HFmrEF): LVEF between 40% and 49%; and HF with preserved EF (HFpEF): LVEF ≥ 50% [6]. Each of these subtypes presents pathophysiological peculiarities, different therapeutic responses and specific prognostic profiles. For example, while HFrEF mainly recognizes systolic dysfunction and responds better to neurohormonal therapy [7], HFpEF is often associated with comorbidities such as hypertension, obesity and diabetes, with a pathophysiological mechanism closer to diastolic dysfunction and a less predictable response to conventional therapy [8]. In parallel with clinical and instrumental characterization, the use of plasma biomarkers in the diagnosis, prognosis and monitoring of the therapeutic response in HF has progressively established itself. Beyond natriuretic peptides (brain natriuretic peptide (BNP) and N-terminal pro-B-type natriuretic peptide (NT-proBNP)), widely validated for diagnosis and risk stratification, increasing attention has been paid to biomarkers that reflect the nutritional, inflammatory and metabolic status of the patient [9]. Among these, serum albumin (SA) is a simple, inexpensive and widely available index, long known for its association with prognosis in several chronic conditions. In HF patients, low albumin levels correlate with an increased risk of adverse events, representing a surrogate marker of malnutrition, chronic inflammation and liver dysfunction [10]. Similarly, high-sensitivity C-reactive protein (hs-CRP), a classic marker of systemic inflammation, has been repeatedly associated with increased cardiovascular risk and poor prognosis in HF patients [11,12,13]. In fact, the inflammatory response represents a central mechanism in disease progression, contributing to ventricular remodeling and endothelial dysfunction [14,15].

A combined approach using the hs-CRP/SA ratio (respectively measured as mg/L and g/dL) has recently been proposed, already validated as a prognostic index in oncology and severe infections [16,17,18]. This ratio simultaneously reflects the patient’s inflammatory and nutritional status, integrating two fundamental dimensions of clinical risk in HF. However, the application of the hsCRP/SA ratio in chronic heart failure remains poorly explored, with few studies available and preliminary results not yet consolidated. In light of these considerations, the present study aims to evaluate the prognostic role of the hs-CRP/SA ratio in predicting major adverse cardiovascular events (MACEs) in a cohort of patients affected by chronic heart failure, contributing to the search for simple, accessible and reliable tools for a more accurate risk stratification.

## 2. Materials and Methods

### 2.1. Study Population

We conducted a retrospective analysis on a cohort of 500 patients with chronic HF, enrolled on a continuous basis, in the period between October 2012 and January 2025, belonging to the Geriatric Department of University Hospital “Renato Dulbecco” of Catanzaro, Italy. All participants provided written informed consent. They were recruited according to the indications of the European Society of Cardiology (ESC) guidelines for the diagnosis and treatment of acute and chronic HF [1]. Inclusion criteria included: age ≥ 18 years; diagnosis of chronic HF according to ESC 2021 criteria [1]; New York Heart association (NYHA) Class II–III; and available high-sensitivity C-reactive protein (hs-CRP) and serum albumin (SA) values at the time of initial assessment. The exclusion criteria included: chronic HF with NYHA class IV; acute HF in the previous 6 months; respiratory failure; severe hepatic impairment (Child–Pugh Class C); severe renal dysfunction (estimated glomerular filtrate (eGFR) < 30 mL/min/1.73 m^2^); nephrotic syndrome; macroalbuminuria; pregnancy or breastfeeding; malnutrition evaluated as Mini Nutritional Assessment (MNA) < 17 pts; and cachexia.

### 2.2. Clinical and Laboratory Data Collection

A detailed clinical history was collected for each patient, with particular attention to comorbidities (arterial hypertension, type 2 diabetes mellitus, ischemic heart disease, atrial fibrillation, chronic obstructive pulmonary disease, chronic kidney disease (CKD), non-alcoholic fatty liver disease (NAFLD). All subjects underwent cardiological examination, blood chemistry tests, echocardiography and measurement of NT-proBNP [19]. High-sensitivity CRP (hs-CRP) values were determined by immunoturbidimetric method (Cardio Phase hs-CRP, Milan, Italy) and was estimated with PATHFAST™ (PHC Corporation, Tokyo, Japan), while serum albumin was measured by colorimetric method (green bromocresol) and was estimated with Alb2 kit on a Cobas C6000 analyzer (Roche Diagnostics, Milan, Italy). The hs-CRP/SA ratio was calculated by dividing the hsCRP (mg/L) by the concentration of albumin (g/dL).

### 2.3. Cardiovascular Endpoints

The primary endpoint of the study was the incidence of a MACE, defined as: nonfatal myocardial infarction, nonfatal ischemic stroke, coronary revascularization (PCI or CABG), or cardiovascular death [20,21,22]. Secondary endpoints included: all-cause mortality; and hospitalizations for HF (hHF). The events were verified through clinical documentation, death certificates, hospital discharge letters or telephone contacts with patients or family members.

### 2.4. Statistical Analysis

The population was divided into two groups based on the median value of the hs-CRP/SA ratio (1.19) in the study population: Group A: hs-CRP/SA < 1.19 (*n* = 249); Group B: hs-CRP/SA ≥ 1.19 (*n* = 251). Continuous variables were expressed as mean ± standard deviation (SD) and compared by *t*-test for independent data; categorical variables such as frequencies and percentages were compared with chi-square tests. The accuracy of the hs-CRP/SA value as a predictor of MACEs, both as a categorical and continuous variable, was evaluated by processing a receiver operating characteristic (ROC) curve. The area under the curve (AUC) described the magnitude to which the hs-CRP/SA value was associated with the onset of events. In addition, we calculated the Youden index (J = Sensitivity + Specificity − 1) to identify the hs-CRP/SA balance threshold value that best balances sensitivity and specificity on the incidence of a MACE. To calculate the incidence of events, number per 100 patients/year was used. Because the follow-up was not uniform for all patients, the onset of a MACE was not assessed at the same time, so a regression analysis based on the Cox proportional model was used, correcting the analysis for variables that differed statistically significantly between the two groups and for variables that could be pathophysiologically associated with the occurrence of MACEs. In particular, a univariate Cox regression model was performed on the incidence of MACEs; subsequently, the variables that significantly correlated with the appearance of MACE were included in a multivariate Cox regression model to calculate the hazard ratio (HR) for the independent predictors associated with the incidence of MACEs. The analysis was corrected for pharmacological treatments. Furthermore, in order to avoid overfitting the model, some variables that did not differ significantly between the two groups but that could correlate with the study endpoint, such as NTproBNP and LVEF, were not included in the Cox regression model. The differences were considered statistically significant for *p* value < 0.05. All analyses were performed using the SPSS v.20.0 statistical program for Windows (SPSS Inc., Chicago, IL, USA).

## 3. Results

### 3.1. Clinical Characteristics of the Population

From an initial cohort of 587 patients, 28 were excluded because they were lost during follow-up, 15 because they had developed acute HF in the previous 6 months, 13 because they had severe CKD, 12 because they had chronic HF NYHA class IV, eight with severe hepatic impairment, seven because they had cachexia, and four because they had respiratory failure (Supplementary Figure S1). Therefore, 500 patients were enrolled including 139 (27.8%) females, with mean ages of 68.6 ± 10.9 years. In terms of decompensation phenotype, 198 (39.6%) had HFrEF, 136 (27.2%) had HFmrEF, and 166 (33.2%) had HFpEF. In addition, significantly higher hs-CRP values were observed in the hsCRP/SA group ≥ 1.19 (5.8 ± 1.3 vs. 3.3 ± 1.1 mg/L; *p* < 0.001) as well as lower serum albumin levels (3.5 ± 0.4 vs. 4.1 ± 0.7 g/dL; *p* < 0.001). Table 1 shows the epidemiological and clinical characteristics of the entire study population, stratified by the clinical cut-off of hs-CRP/SA (1.19). Statistically significant differences between the two groups were observed for prevalence of Ischemic Heart Disease and NAFLD, significantly higher in patients with hs-CRP/SA > 1.19. There was no difference between the two groups for intake of antihypertensive drugs, insulin, oral anticoagulants, anti-aggregants, statins, beta-blockers, loop diuretics, sacubitril–valsartan and mineralocorticoid receptor antagonists, while there were differences for intake of GLP1-RA. Regarding clinical, hemodynamic, and laboratory parameters, there were statistically significant differences between the two groups for HbA1c, hsCRP, uricemia, E/e’ and s-PAP, significantly higher in patients with hsCRP/SA > 1.19 (Table 1). Meanwhile, Hct, LDL, SA, Transferrin, LVEF and tricuspid annular plane systolic excursion (TAPSE) were significantly higher in patients with hs-CRP/SA < 1.19 (Table 1).

### 3.2. Incidence of Cardiovascular Events and Mortality

**Figure 1:**
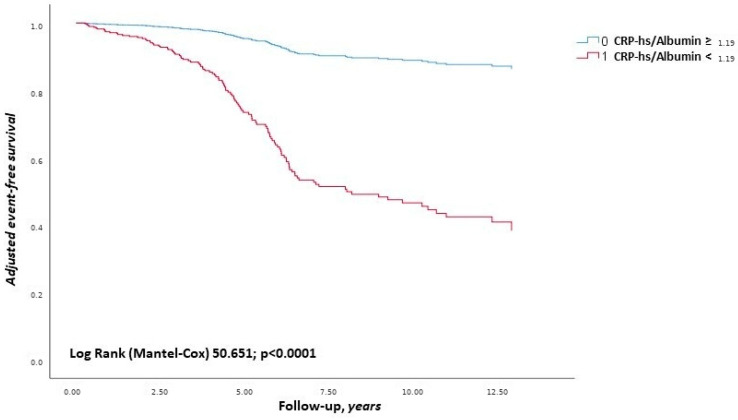
Adjusted Kaplan–Meier on MACEs, according to cut-off value of hs-CRP/SA. Adjusted for: SA as dichotomous value, hs-CRP as dichotomous value, IHD, UA as dichotomous value, CKD, and age as 10 years. Abbreviations: MACE, major adverse cardiovascular event; SA, serum albumin; IHD, ischemic heart disease; UA, uric acid; CKD, chronic kidney disease.

During a median follow-up of 5.2 years, 115 MACEs occurred, corresponding to an incidence of 3.6 events per 100 patient-years (Figure 1). The incidence was significantly higher in the hs-CRP/SA group ≥ 1.19 than in the hs-CRP/SA group < 1.19 (5.9 vs. 1.2 events/100 patient-years; *p* < 0.001) (Table 2). The incidence of a MACE in the three HF phenotypes was calculated as follows: 36/162 in the HFrEF group (22.2%), 44/92 in the HFmrEF group (47.8%), and 35/131 in the HFpEF group (26.7%).

**Figure 2:**
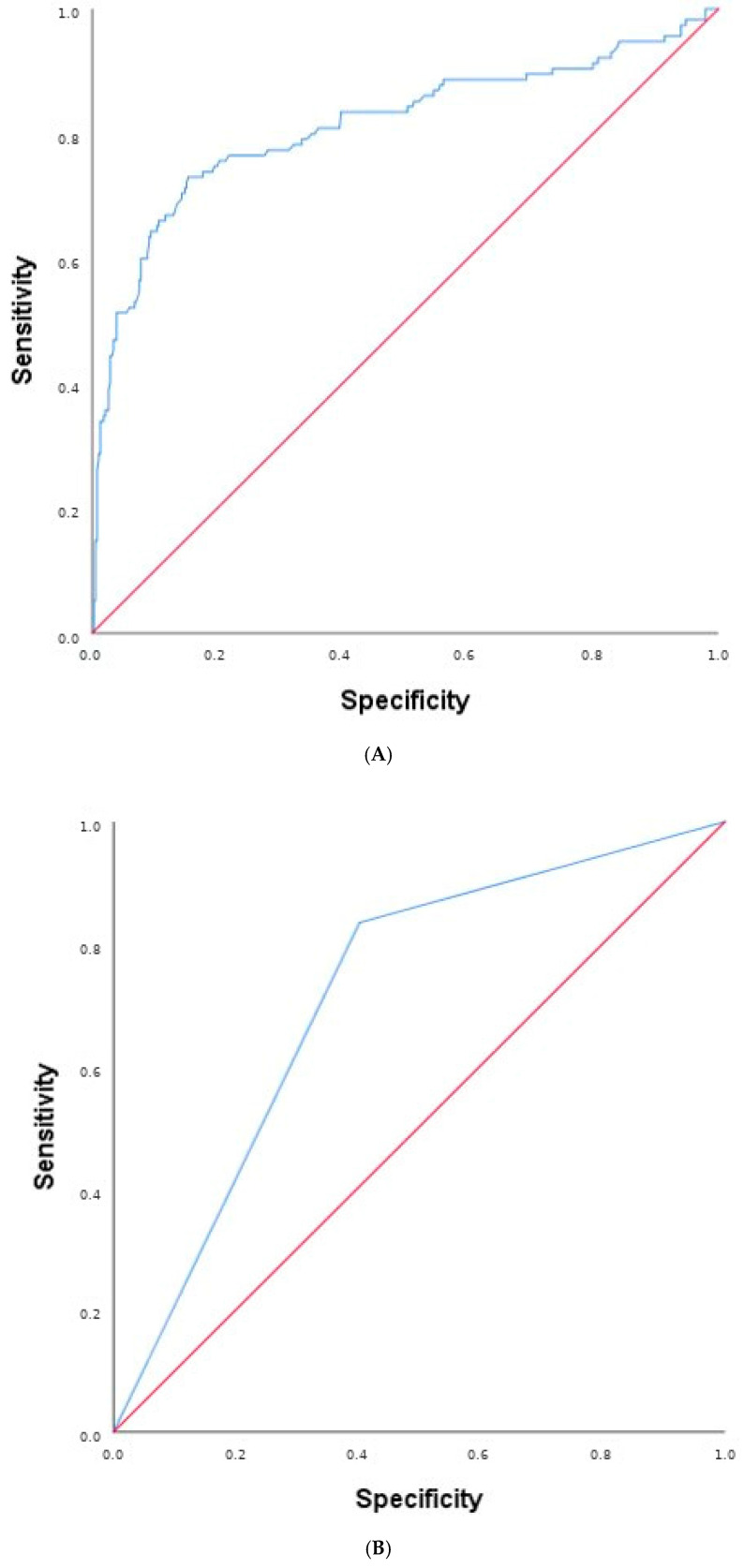

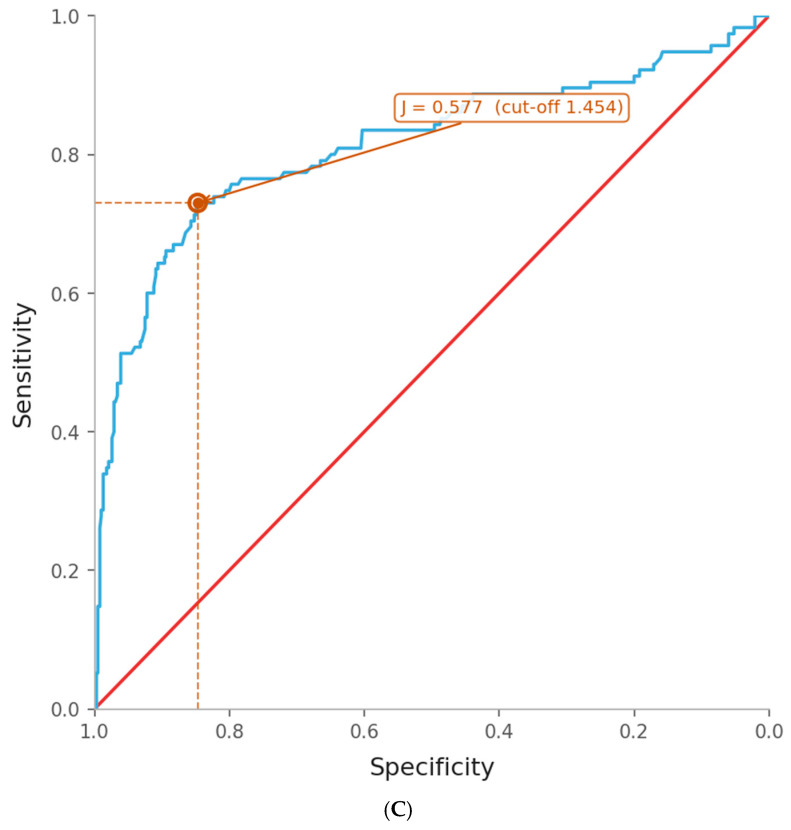
(**A**) ROC curves on MACEs, according to hs-CRP/SA as continuous variable. (**B**)—ROC curves on MACEs, according to hs-CRP/SA as dichotomous variable. (**C**) ROC curves on MACEs, according to hs-CRP/SA as continuous variable with Youden’s index. Abbreviations: MACE, major cardiovascular adverse event; ROC, receiver operating characteristic; hs-CRP, high-sensitivity C-reactive protein; SA, serum albumin.

Differences in the individual components of MACEs included: nonfatal myocardial infarction (3.3 vs. 0.3 events/100 patient-years (*p* < 0.001)), nonfatal ischemic stroke (1.4 vs. 0.4 events/100 patients/year (*p* = 0.004)) and cardiovascular mortality (1.3 vs. 0.4 events/100 patients/year (*p* = 0.007)) (Table 2). Total mortality (3.6 vs. 1.6 events/100 patient-years; *p* < 0.001) and HF hospitalizations (6.5 vs. 2.9 events/100 patient-years; *p* < 0.001) were also significantly higher in the group with high hs-CRP/SA. (Table 2). The accuracy of hs-CRP/SA as a predictive value of the onset of MACEs both as a continuous (Figure 2A) and as a dichotomous value (Figure 2B) was evaluated by the AUC. Figure 2A shows the ROC curve of hs-CRP/SA as a continuous variable; notably, hs-CRP/SA as a continuous variable has a greater discriminating power in predicting the development of MACEs (AUC 0.813; standard error 0.027; 95% CI 0.760–0.867; *p* < 0.001), compared with hs-CRP/SA as a dichotomous value (AUC 0.716; standard error 0.026; 95% CI 0.666–0.767; *p* < 0.001) (Figure 2B). Furthermore, with a Youden index of 0.577, the hs-CRP/SA value of 1.454 was identified as the threshold that best balances sensitivity and specificity, minimizing both false negatives and false positives (Figure 2C): sensitivity 73.0%, specificity 84.7%, negative predictive value (NPV) 91.3%, and positive predictive value 58.7%.

### 3.3. Regression Analysis

Cox’s univariate analysis identified the hs-CRP/SA ratio ≥ 1.19 as strongly associated with an increased risk of a MACE (HR 7.213; 95% CI 4194–12,404; *p* < 0.001). Other significantly associated factors included: age (HR for every 10 years: 1.282; *p* = 0.010), presence of CKD (HR 2.595; *p* < 0.001), higher uricemia (HR 0.864 for every 1 mg/dL; *p* = 0.047), and absence of treatment with SGLT2i or statins (Table 3).

In the stepwise multivariate model, the hs-CRP/SA ratio ≥ 1.19 remained an independent predictor of MACEs (HR 6.513; 95% CI 3928–10,797; *p* < 0.001). In addition, SGLT2i use (HR 0.382; *p* < 0.001), statin use (HR 0.438; *p* = 0.007), and uricemia reduction (HR 0.839 for every 1 mg/dL; *p* = 0.013) were independently associated with risk reduction in MACEs. On the contrary, the presence of CKD (HR 2.350; *p* < 0.001) and the increase of 10 years of age (HR 1.249 for every 10 years; *p* = 0.009) were confirmed to be associated with a higher risk of events (Table 4). We also performed a subgroup analysis, which showed that, in HFrEF patients, an hS-CRP value ≥ 1.19 is associated with a 6.81-fold increase in the incidence of MACEs (HR 6.818, CI 95% 2.409–19.29, *p* < 0.0001); in patients affected by HFmrEF by 3.43 times (HR 3.435, CI 95% 1.650–7.151, *p* = 0.001); in those with HFpEF of 6.98 times (HR 6.989, CI 95% 2.900–16.845, *p* < 0.0001); therefore, the association between hs-CRP values ≥ 1.19 and MACE incidence persists in all three HF phenotypes.

## 4. Discussion

In the present retrospective study of 500 outpatients with chronic heart failure, the high-sensitivity C-reactive protein/serum albumin (hsCRP/SA) ratio proved to be a powerful independent predictor of MACEs during a long-term follow-up of over 5 years. In particular, a hs-CRP/SA value ≥ 1.19 was associated with a more than six-fold increased risk of a MACE, confirming its prognostic role even after adjustment for comorbidities and pharmacological treatments. Our results extend the evidence already available on the use of albumin as a prognostic biomarker in heart failure [23,24,25]. Previous studies, including that of Armentaro et al. (2024) [23], had demonstrated that low albumin levels (<3.5 g/dL) are associated with an increase in MACEs and mortality. However, this new work introduces a significant innovation: the integration of albumin with hs-CRP, a highly sensitive inflammatory marker, improves the ability to discriminate risk. In fact, the hs-CRP/SA ratio reflects both the inflammatory state and the nutritional reserve, two central determinants of prognosis in patients with heart failure. From a pathophysiological point of view, it is known that chronic inflammation plays a key role in the progression of HF and in the development of atherothrombotic events [26,27,28]. Hs-CRP not only reflects the systemic inflammatory state, but also promotes endothelial dysfunction, platelet activation and the destabilization of atherosclerotic plaques [29,30,31]. On the other hand, albumin exerts antioxidant, anti-inflammatory and antithrombotic effects: its reduction can alter the hemostatic balance and promote cardiovascular events. The hs-CRP/SA ratio, therefore, could represent a synthetic indicator of the biological vulnerability of the patient, particularly in the presence of chronic cardiovascular diseases.

Several studies have evaluated the potential role of hs-CRP/SA as a prognostic factor in patients with HF. In particular, in patients with acute HF, after a 46-month follow-up, an elevated hs-CRP/SA ratio was associated with an increased risk of all-cause mortality [32]. Furthermore, even in patients with chronic HF, this biomarker has been shown to be significantly associated with advanced NYHA class and worse hemodynamic status [33]. However, none of these studies evaluated the possible association of hs-CRP/SA with the incidence of MACEs in a population of patients predominantly affected by chronic HF in NYHA class II-III over a long follow-up period.

A further study evaluated the potential prognostic role of CRP/SA in a cohort of patients with chronic HF, and this study showed that this biomarker had a 73% sensitivity in predicting overall mortality. Furthermore, this finding was confirmed for increasing quartiles of CSP/SA, where mortality was higher than in other quartiles. However, in this study, the mean age was slightly lower than that of the patients enrolled in our study (66.4 ± 1.1 vs. 68.6 ± 10.9 years), and they had a lower burden of comorbidities compared to the population enrolled in our study, such as type 2 diabetes (28.5% vs. 48.6%), arterial hypertension (55.2% vs. 59.4%), and IHD (51.8% vs. 58.8%) [34].

Interestingly, in our multivariate model, the use of sodium–glucose cotransporter 2 inhibitors (SGLT2i) and statins was associated with a statistically significant reduction in the risk of a MACE, suggesting a direct protective role of these therapies in patients with heart failure. SGLT2i have already shown a solid efficacy in reducing hospitalizations and cardiovascular mortality, both in patients with reduced and preserved ejection fraction [34]. These benefits, documented in trials such as DAPA-HF, EMPEROR-Reduced and more recently DELIVER, also extend to non-diabetic patients [35,36,37,38]. The mechanisms of action include reduction in extracellular volume, renal protection, neurohormonal modulation and improvement of cardiac energy efficiency. These effects contribute directly to the prevention of major events, regardless of the baseline inflammatory profile [39,40]. Statins, for their part, continue to demonstrate a key role in secondary prevention in patients with known cardiovascular disease. In addition to their lipid-lowering effect, they stabilize the atherosclerotic plaque and improve endothelial function, helping to reduce the risk of myocardial ischemia, stroke and sudden death [41,42]. In our study, their use is also associated with a lower rate of MACEs, confirming the value of these therapies in a high-risk population such as that with chronic HF. Similarly, the reduction in serum uric acid levels was confirmed as an additional factor associated with a reduced risk of MACEs, in line with data identifying hyperuricemia as a biomarker of oxidative stress and inflammation in patients with HF [43].

Furthermore, considering that in this study, the value of hs-CRP/SA < 1.454 has a high NPV (91.3%), this means that they have a high probability of not developing the event. In particular, this biomarker could represent a composite indicator of systemic inflammation, malnutrition, and overall frailty, which is associated with the incidence of a MACE in a population of patients with chronic HF and numerous comorbidities. Like other observational studies, ours also has some limitations. First, the retrospective nature does not allow the establishment of causal relationships. Furthermore, hs-CRP and albumin were measured only once at baseline; therefore, we cannot assess the impact of longitudinal changes in hs-CRP/SA over time, and the dichotomization of the biomarker may have led to an overestimation of the effect. A further limitation is the single-center design and possible heterogeneity of medical therapy in the two groups, despite multivariate adjustment. Future studies should incorporate head-to-head comparisons with NT-proBNP, troponins, or other established markers to clarify the ratio’s unique contribution to risk stratification. Finally, our cohort was predominantly composed of NYHA class II-III patients, so the applicability of the results to patients with more advanced or unstable HF may be limited.

## 5. Conclusions

The present study demonstrates that the hs-CRP/SA is associated with the incidence of major adverse cardiovascular events (MACEs) in outpatients with chronic HF. A hs-CRP/SA value ≥ 1.19 is associated with an increased risk of a MACE, regardless of age, renal comorbidities, inflammation and drug treatment. Integrating hs-CRP/SA, which is easily obtained in routine clinical assessment, could significantly improve prognostic stratification and support more effective personalization of treatment in HF patients. In fact, taking into account inflammatory and nutritional parameters, it could indicate future treatment targets in high-risk patients who are already on top of therapy. Further prospective, multicenter studies and external validation in prospective cohorts will be needed to confirm these findings and evaluate the dynamic predictive value of hs-CRP/SA in long-term monitoring of HF patients.

## Figures and Tables

**Table 1:** Clinical, epidemiological, laboratory, echocardiographic parameters and pharmacotherapy of study population at baseline according to clinical cut-off of CRP/SA.

	All Population (*n*. 500)	hs-CRP/SA < 1.19 (*n*. 249)	hs-CRP/SA ≥ 1.19 (*n*. 251)	*p* *
Age, years	68.6 ± 10.9	69.6 ± 10.7	67.6 ± 11.1	0.050 ^ǂ^
Female sex, *n* (%)	139 (27.8)	64 (12.8)	75 (15.0)	0.297 *
HFrEF, *n* (%)	198 (39.6)	88 (17.6)	110 (22.0)	0.052 *
HFmrEF, *n* (%)	136 (27.2)	63 (12.6)	73 (14.6)	0.342 *
HFpEF, *n* (%)	166 (33.2)	98 (19.6)	68 (13.6)	0.004 *
IHD, *n* (%)	294 (58.8)	135 (27.0)	159 (31.8)	0.038 *
Arterial hypertension, *n* (%)	297 (59.4)	148 (29.6)	149 (29.8)	0.986 *
AF, *n* (%)	124 (24.8)	70 (14.0)	54 (10.8)	0.088 *
Dislipidemia, *n* (%)	225 (45.0)	125 (25.0)	100 (20.0)	0.020 *
SAS, *n* (%)	164 (32.8)	82 (16.4)	82 (16.4)	0.950 *
CKD, *n* (%)	140 (28.0)	64 (12.8)	76 (15.2)	0.255 *
COPD, *n* (%)	106 (21.2)	51 (10.2)	55 (11.0)	0.696 *
NAFLD, *n* (%)	53 (10.6)	15 (3.0)	38 (7.6)	<0.001 *
Obesity, *n* (%)	130 (26.0)	65 (13.0)	65 (13.0)	0.958 *
T2DM, *n* (%)	243 (48.6)	120 (24.0)	123 (24.6)	0.856 *
Alcohol, *n* (%)	63 (12.6)	32 (6.4)	31 (6.2)	0.866 *
Smokers, *n* (%)	147 (29.4)	64 (12.8)	83 (16.6)	0.071 *
MLHFQ, pt	95.93 ± 3.3	95.9 ± 3.4	95.9 ± 3.1	0.860 ^ǂ^
SBP, mmHg	123.1 ± 15.3	123.1 ± 15.0	123.1 ± 15.6	0.986 ^ǂ^
DBP, mmHg	74.5 ± 9.6	74.5 ± 9.6	74.5 ± 9.6	0.996 ^ǂ^
HR, bfm	69.7 ± 14.0	69.3 ± 14.0	70.2 ± 14.1	0.521 ^ǂ^
RR, afm	17.6 ± 2.6	17.0 ± 2.7	18.3 ± 2.4	0.745 ^ǂ^
Hb, g/dL	12.7 ± 1.9	12.9 ± 1.9	12.6 ± 1.9	0.070 ^ǂ^
Hct, %	39.5 ± 5.5	40.1 ± 5.7	38.9 ± 5.2	0.015 ^ǂ^
Na, mmol/L	140.7 ± 2.7	140.7 ± 2.5	140.7 ± 2.8	0.841 ^ǂ^
Mg, mg/dL	1.9 ± 0.2	1.96 ± 0.1	1.94 ± 0.2	0.225 ^ǂ^
K, mmol/L	4.4 ± 0.44	4.4 ± 0.4	4.4 ± 0.4	0.360 ^ǂ^
HbA1c, %	6.5 ± 0.8	6.4 ± 0.8	6.6 ± 0.7	0.049 ^ǂ^
eGFR, mL/min/1.73 m^2^	70.1 ± 19.6	70.1 ± 19.6	70.1 ± 19.7	0.991 ^ǂ^
Microalbuminuria, mg/L	34.2 ± 14.2	41.4 ± 17.4	29.3 ± 8.6	0.498 ^ǂ^
LDL, mg/dL	67.4 ± 32.4	70.4 ± 31.6	64.4 ± 33.0	0.037 ^ǂ^
Triglycerides, mg/dL	124.2 ± 50.1	126.5 ± 53.1	121.9 ± 46.8	0.308 ^ǂ^
AST, U/L	21.6 ± 9.0	21.5 ± 9.2	21.8 ± 8.7	0.691 ^ǂ^
ALT, U/L	21.4 ± 9.5	21.5 ± 9.6	21.3 ± 9.4	0.835 ^ǂ^
γ-GT, U/L	32.8 ± 18.7	31.4 ± 18.3	34.2 ± 19.1	0.095 ^ǂ^
Albumin, mg/dL	3.8 ± 0.6	4.1 ± 0.7	3.5 ± 0.4	<0.001 ^ǂ^
NT-pro-BNP, pg/mL	2253.4 ± 769.7	2169.8 ± 851.3	2316.6 ± 697.3	0.076 ^ǂ^
Uricemia, mg/dL	5.7 ± 1.4	5.5 ± 1.4	5.8 ± 1.3	0.032 ^ǂ^
Transferrin, mg/dL	215.9 ± 55.4	224.3 ± 56.4	207.5 ± 53.17	<0.001 ^ǂ^
Ferritin, ng/mL	187.3 ± 71.0	187.0 ± 75.0	187.6 ± 67.0	0.920
hs-CRP, mg/L	4.6 ± 1.7	3.3 ± 1.1	5.8 ± 1.3	<0.001 ^ǂ^
LAVi, mL/m^2^	45.1 ± 12.2	46.0 ± 13.1	44.3 ± 11.2	0.117 ^ǂ^
LVEF, %	44.7 ± 9.1	45.8 ± 9.2	43.6 ± 8.9	0.007 ^ǂ^
CI, mL/min/1.73 m^2^	1907.1 ± 205.9	1906.6 ± 219.5	1907.7 ± 192.3	0.958 ^ǂ^
E/A	1.01 ± 0.51	1.07 ± 0.49	0.94 ± 0.52	0.011 ^ǂ^
E/e′	14.9 ± 4.5	14.4 ± 4.4	15.4 ± 4.7	0.018 ^ǂ^
TAPSE, mm	18.7 ± 4.1	19.1 ± 4.2	18.4 ± 4.0	0.043 ^ǂ^
s-PAP, mmHg	40.5 ± 12.0	39.3 ± 11.8	41.7 ± 12.0	0.027 ^ǂ^
TAPSE/s-PAP, mm/mmHg	0.51 ± 0.23	0.54 ± 0.23	0.49 ± 0.23	0.020 ^ǂ^
RVOTp	2.75 ± 0.40	2.75 ± 0.40	2.75 ± 0.39	0.902 ^ǂ^
Area atrio dx	19.47 ± 4.37	19.78 ± 5.07	19.15 ± 3.52	0.107 ^ǂ^
β-blockers, *n* (%)	395 (79.0)	190 (38.0)	205 (41.0)	0.141 *
MRAs, *n* (%)	194 (38.8)	94 (18.8)	100 (20.0)	0.632 *
RAASi, *n* (%)	459 (91.8)	226 (45.2)	233 (46.6)	0.400 *
SGLT2i, *n* (%)	193 (38.6)	88 (17.6)	105 (21.0)	0.136 *
Loop diuretics, *n* (%)	364 (72.8)	175 (35.0)	189 (37.8)	0.231 *
GLP1-RAs, *n* (%)	74 (14.8)	47 (9.4)	27 (5.4)	0.013 *
OADs, *n* (%)	184 (36.8)	92 (18.4)	92 (18.4)	0.231 *

* Performed by chi-square test. ^ǂ^ Performed by *t*-test for unpaired data. Abbreviations: HFrEF: heart failure with reduced ejection fraction; HFmrEF: heart failure with mid-range fraction; HFpEF: heart failure with preserved ejection fraction; IHD: ischemic heart disease; AF: atrial fibrillation; SAS: sleep apnea syndrome; CKD: chronic kidney disease; COPD: chronic obstructive pulmonary disease; NAFLD: non-alcoholic fatty liver disease; T2DM: type 2 diabetes mellitus; MLHFQ: Minnesota Living with Heart Failure Questionnaire; SBP: systolic blood pressure; DBP: diastolic blood pressure; HR: heart rate; RR: respiratory rate; Hb: hemoglobin; Hct: hematocrit, Na: Sodium; Mg: Magnesium; K: Potassium; HbA1c: glycated hemoglobin; e-GFR: estimate glomerular filtration rate; LDL: low density lipoprotein; AST: aspartate aminotransferase; ALT: alanine aminotransferase; γ-GT: gamma-glutamyltransferase; NT-pro-BNP: N-terminal pro-brain natriuretic peptide; hs-CRP: high-sensitivity C-reactive protein; LAVi: left atrial volume index; LVEF: left ventricular ejection fraction; CI: cardiac index; RVOTp: right ventricular outflow tract proximal; E/A: ratio between wave E (the wave of rapid filling in early diastole) and wave A (the wave of atrial contraction); E/e′: between wave E and wave e′ (reliable estimate of changes in end-diastolic blood pressure); TAPSE: tricuspid annular plane systolic excursion; s-PAP: systolic pulmonary arterial pressure; MRAs: mineralocorticoid receptor antagonists; RAASi: renin–angiotensin–aldosterone system inhibitors; SGLT2i: sodium–glucose cotransporter 2 inhibitor; GLP1-RAs: glucagon-like peptide 1 receptor agonists; OADs: oral antidiabetic drugs.

**Table 2:** Cardiovascular events and hHF in the study population according to clinical cut-off of CRP/SA.

	All Population (*n*. 500)	hs-CRP/SA < 1.19 (*n*. 249)	hs-CRP/SA ≥ 1.19 (*n*. 251)	*p*
MACE, *n* (%)	115 (3.6)	19 (1.2)	96 (5.9)	<0.001 *
Nonfatal Stroke, *n* (%)	29 (0.9)	7 (0.4)	22 (1.4)	0.004 *
NF coronary events, *n* (%)	58 (1.8)	5 (0.3)	53 (3.3)	<0.001 *
CV mortality, *n* (%)	28 (0.9)	7 (0.4)	21 (1.3)	0.007 *
Non-CV mortality, *n* (%)	55 (1.7)	19 (1.2)	36 (2.2)	0.016 *
Total mortality, *n* (%)	83 (2.6)	26 (1.6)	57 (3.6)	<0.001 *
hHF, *n* (%)	152 (4.8)	47 (2.9)	105 (6.5)	<0.001 *

* Performed by chi-square test. Data described as number of patients (number of events per 100 patient-years). Abbreviations: hs-CRP: high-sensitivity C-reactive protein; hHF: heart failure hospitalizations; MACE: major adverse cardiovascular event; NF: nonfatal; CV: cardiovascular.

**Table 3:** Univariate Cox regression analysis on MACEs.

	HR	95% CI	*p*
Female sex, yes/no	0.942	0.614–1.447	0.786
Age, 10 years	1.282	1.062–1.549	0.010
IHD, yes/no	1.578	0.988–2.520	0.056
AF, yes/no	1.008	0.601–1.690	0.976
Dyslipidemia, yes/no	1.112	0.746–1.660	0.602
CKD, yes/no	2.595	1.713–3.932	<0.001
COPD, yes/no	0.920	0.572–1.479	0.730
NAFLD, yes/no	1.272	0.767–2.107	0.351
Beta-Blockers, yes/no	0.736	0.426–1.273	0.273
RAASi, yes/no	1.009	0.402–2.535	0.985
MRA, yes/no	0.850	0.564–1.280	0.436
SGLT2i, yes/no	0.341	0.181–0.640	0.001
GLP1-RAs, yes/no	0.955	0.567–1.608	0.861
Statins, yes/no	0.361	0.186–0.700	0.003
HbA1c > 6.8%, yes/no	0.789	0.579–1.076	0.135
UA, mg/dL	0.864	0.748–0.998	0.047
Transferrin, mg/dL	1.000	0.996–1.004	0.910
hs-CRP/SA ≥ 1.19, yes/no	7.213	4.194–12.404	<0.001
LVEF, %	0.996	0.964–1.029	0.805
E/e′	0.986	0.937–1.037	0.571
TAPSE, mm	0.955	0.906–1.007	0.090
s-PAP, mmHg	0.988	0.968–1.008	0.226

Abbreviations: MACE: major adverse cardiovascular event; IHD: ischemic heart disease; AF: atrial fibrillation; CKD: chronic kidney disease; COPD: chronic obstructive pulmonary disease; NAFLD: non-alcoholic fatty liver disease; RAASi: renin–angiotensin–aldosterone system inhibitors; MRA: mineralocorticoid receptor antagonists; SGLT2i: sodium–glucose cotransporter 2 inhibitors; GLP1-RAs: GLP-1 receptor agonists; HbA1c: glycated hemoglobin; UA: uric acid; hs-CRP/SA: high-sensitivity C-reactive protein/serum albumin; LVEF: left ventricular ejection fraction; E/e′: between wave E and wave e′ (reliable estimate of changes in end-diastolic blood pressure); TAPSE: tricuspid annular plane systolic excursion; s-PAP: systolic pulmonary arterial pressure.

**Table 4:** Multivariate stepwise Cox regression analysis on MACEs.

	HR	CI 95%	*p*
hs-CRP/SA ≥ 1.19, yes/no	6.513	3.928–10.797	<0.001
SGLT2i, yes/no	0.382	0.246–0.594	<0.001
Statins, yes/no	0.438	0.251–0.766	0.007
UA, 1 mg/dL reduction	0.839	0.727–0.968	0.013
CKD, yes/no	2.350	1.597–3.456	<0.001
Age, 10 years increase	1.249	1.054–1.480	0.009

Abbreviations: MACE: major adverse cardiovascular event; hs-CRP: high-sensitivity C-reactive protein; SGLT2i: sodium–glucose cotransporter 2 inhibitors; UA: uric acid; CKD: chronic kidney disease.

## Data Availability

The data underlying this article will be shared upon reasonable request to the corresponding author.

## Supplementary Materials

The following supporting information can be downloaded at https://www.mdpi.com/article/10.3390/biomedicines14040748/s1, Figure S1: Flowchart of patients enrolled in the study.
