# Supplementary figures and images for: Prognostic Role of High-Sensitivity C-Reactive Protein/Albumin Ratio in Heart Failure Patients
Source: Biomedicines. 2026 Mar 25;14(4):748. doi: 10.3390/biomedicines14040748 (PMC13114191; doi:10.3390/biomedicines14040748)

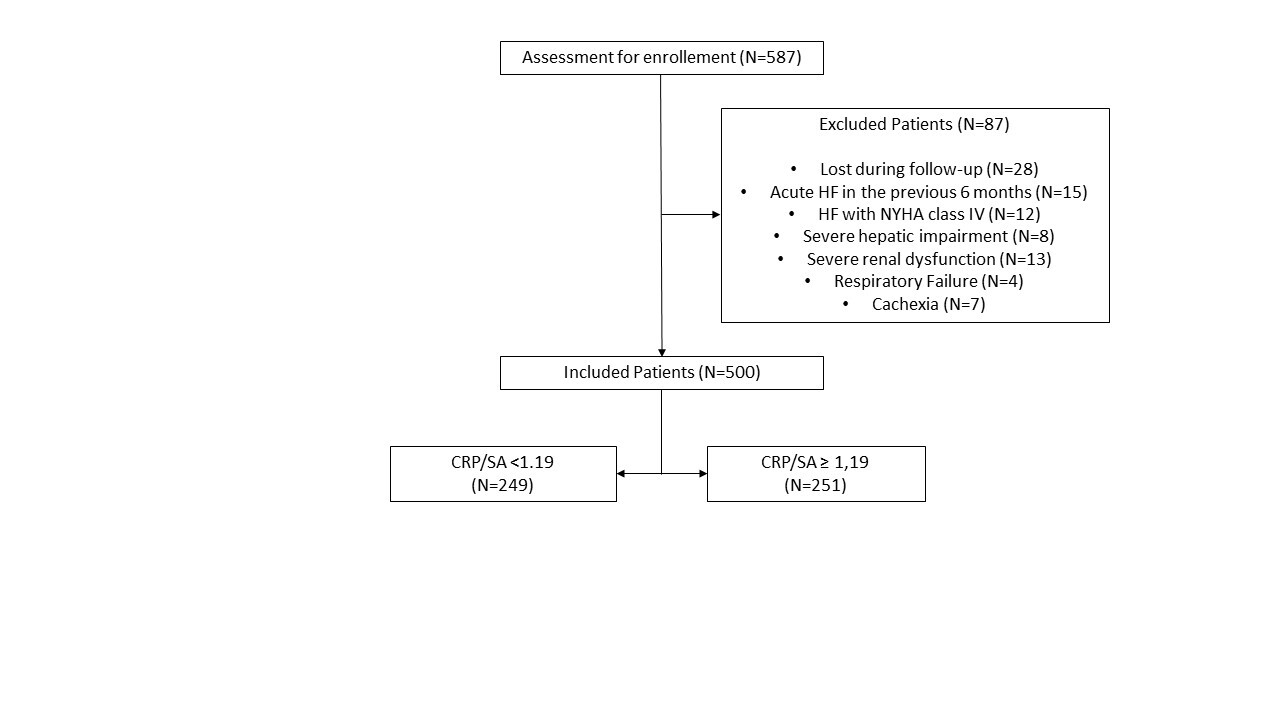

Supplement: Supplementary file 1 [file biomedicines-14-00748-s001.zip › biomedicines-4175369-supplementary-Figure S1.jpg]
